# Chip-fiber-chip quantum teleportation in a star-topology quantum network

**DOI:** 10.1038/s41377-025-02034-2

**Published:** 2025-10-06

**Authors:** Anahita Khodadad Kashi, Michael Kues

**Affiliations:** 1 Institute of Photonics, Leibniz University Hannover, 30167 Hannover, Germany; 2 Cluster of Excellence PhoenixD (Photonics, Optics, Engineering—Innovation Across Disciplines), Leibniz University Hannover, 30167 Hannover, Germany

**Keywords:** Integrated optics, Quantum optics

## Abstract

A recent research reports on chip-fiber-chip quantum teleportation of time-bin-encoded qubits over a 12.3 km optical fiber link within a star-topology quantum network, composed of an on-chip accommodated user node, relay node and a central node. An active feedback optimization scheme is embedded to ensure highly stable Bell state measurements.

Scalability of quantum networks is essential to the future realization of the quantum internet^1^.

To efficiently expand the network size, preserving the coherence of quantum states is essential. As quantum networks scale, decoherence, noise, and optical loss, inevitably restrict the distance range for direct transmission of quantum states via physical channels, such as free space and optical fibers. This underscores the significance of embedding relay nodes in large-scale quantum networks to mediate the transmission of quantum states via quantum teleportation^2^, which involves transferring an unknown quantum state through a previously shared entanglement link, critical for e.g., realizing distributed quantum computing^3^. Teleportation rate, fidelity, and high quality entanglement distribution range are essential metrics for assessing the scalability of quantum communication protocols^4^.

In free-space quantum communications, a major breakthrough was achieved with the quantum teleportation of an unknown polarization-encoded quantum state over a distance of up to 1400 km via an uplink channel to the low-orbit Micius satellite^5^. For fiber-optic quantum communications in metropolitan scale, time-bin encoding is particularly well-suited due to its intrinsic robustness to polarization drift in optical fibers. Quantum teleportation of time-bin decoy states using table-top/fiber-based optical telecommunication infrastructure was demonstrated at a teleportation rate of 7.1 ± 0.4 Hz over 64 km of optical fiber link^6^ and quantum teleportation of time-bin single-photon states was reported over 44 km single-mode optical fiber at a few Hz teleportation rate with a fidelity above 90%^7^.

However, pushing forward the scalable deployment of quantum networks requires in parallel the miniaturization of system components for mass-reproducible chip-scale components. Chip-to-chip quantum teleportation has been demonstrated using path-polarization converters, however limited to 10 m long fiber link^8,9^. Further advancements are thus required in the field to extend the distance of chip-fiber-chip quantum teleportation.

In a recent publication^10^, researchers have proposed a scalable chip-based star-topology quantum network design via a relay node configuration, and have demonstrated chip-fiber-chip quantum teleportation of time-bin-encoded single-photon states, showcasing an integrated approach for extending the effective distance range in quantum networks. In this work, on a packaged silicon photonic chip, three quantum circuits are integrated (see Fig. 1), corresponding to a user node (Alice), a relay node (Charlie), and a central node (Bob). In the user node, from pulsed-excited spontaneous four-wave mixing (SFWM) process, an unknown heralded time-bin-encoded quantum state $\left|{\phi }\right\rangle =\alpha \left|e\right\rangle +\beta {e}^{i\varphi }\left|l\right\rangle$ is prepared ($\left|e\right\rangle$ and $\left|l\right\rangle$ denoting the early and late time-bins, respectively) and directly transmitted through a 6.15 km-long optical fiber link to the relay node. The same pulsed laser source is used for the generation of a time-bin entangled quantum state $\left|{\Psi }^{\pm }\right\rangle =\frac{1}{\sqrt{2}}(\left|{e}_{s},{e}_{i}\right\rangle +{e}^{i\theta }\left|{l}_{s},{l}_{i}\right\rangle )$ from SFWM in the relay node. One photon of an entangled pair is transmitted to the central node via a 6.15 km long optical fiber, establishing an a priori correlation/entanglement link between the relay node and the central node. The other photon of the entangled pair remains in the relay node to be used for the joint Bell state measurement with the qubit sent from the user node, thereby teleporting the quantum state $\left|{\phi }\right\rangle$ to the central node. Projective measurements are performed in the central node to recover the quantum state $\left|{\phi }\right\rangle$ and to characterize its fidelity through quantum state tomography. An active feedback system based on tracking the deviations of the Hong-Ou-Mandel interference visibility has been adopted to stabilize the Bell state measurements in the relay node.

**Fig. 1:**
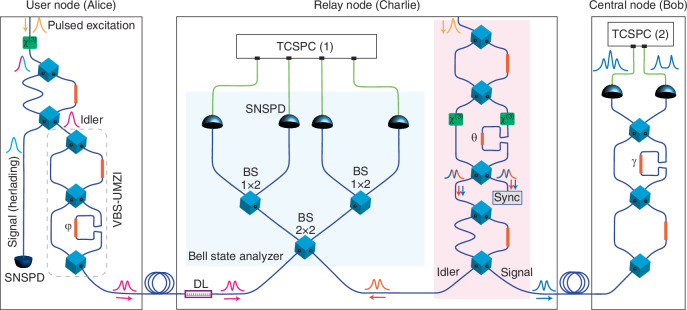
Simplified schematic of the star-topology quantum network. The user node, relay node and the central node are implemented on a single silicon photonic chip. A trigger signal from the pulsed laser source is employed for the synchronization of the TCSPC (1) at the relay node and another trigger signal from the pulse laser is encoded on an optical carrier and sent to the central node for synchronization with the TCSPC (2) at the central node. An optical delay line is used between the user node and the relay node in order to temporally match the arrival time of the idler photons from the user node and the relay node. The visibility of the Hong-Ou-Mandel interference between two thermal fields is used as an error signal for the delay adjustment. The heralding signal in the user node is encoded on an optical carrier and employed in the active stabilization system of the two-photon interference. In the relay node, the signal and idler photons from one output port of the VBS-UMZI are used for synchronization of the user node and the relay node. In this schematic, the blue and green solid lines are the optical and electronic paths in the setup, respectively. (BSM Bell state measurement, BS beam splitter, VBS-UMZI variable beam splitter unbalanced Mach-Zehnder interferometer, ${\chi }^{(3)}$ third-order nonlinear source, TCSPC time-correlated single-photon counter, Sync synchronization signal, DL delay line)

Overall, the authors have demonstrated quantum teleportation of an unknown quantum state over a 12.3 km optical fiber link with an average fidelity of >81% achieved for the recovered states, falling perfectly above the classical limit of 2/3, which indicates a successful implementation of quantum teleportation. With this approach, the direct transmission distance of photons from the user node to the central node is reduced, allowing to efficiently expand the network size without compromising the entanglement quality. Importantly, in this research, fully on-chip implementation of essential steps in quantum teleportation has been realized, paving the path towards scalable, stable, and reproducible development of quantum networks.

The relatively low teleportation rate of 0.01 Hz in this work is outweighed by its larger range of distance, which renders it directly deployable for metropolitan-scale quantum communications. Further integration of excitation laser, delay lines and modulator as well as eventually the stabilization electronics^11^ would be subject to future development.

To address network scalability, leveraging high-dimensional quantum states would allow for enhanced quantum teleportation rates. Using hybrid polarization-path approach for Bell state measurements, quantum teleportation of path-encoded qutrits was demonstrated, however, using free-space setups^12^. In combination with high-dimensionality, quantum teleportation of hyper-entangled quantum states would allow to further extend the distance of quantum communications. Specifically, quantum teleportation of hyper-entangled quantum states in spin- and orbital-angular momentum degrees-of-freedom has been reported^13^. Nevertheless, the stringent requirements on phase stability, polarization drift compensation requirements, and the nonlinear rate of hardware overhead inevitably restrict the scalability of these bulk-scale implementations.

In perspective, complex high-dimensional quantum states using discrete time^14^ and frequency^15^ degrees-of-freedom in photonic chip-integrated platforms, could facilitate scalable quantum teleportation for global-scale quantum communication networks. By harnessing the compatibility of photonic time/frequency degree-of-freedom with standard adaptive telecommunication components, such as reconfigurable wavelength de/multiplexers^16,17^, and by developing efficient on-chip time/frequency manipulation techniques^18^, the scalable implementation of quantum teleportation with performance metrics capable of meeting modern communication demands in terms of transmission rate and scalability becomes feasible.
